# Skeletal Muscle Cells Generated from Pluripotent Stem Cells

**DOI:** 10.1155/2017/7824614

**Published:** 2017-09-24

**Authors:** Yuko Miyagoe-Suzuki, Atsushi Asakura, Masatoshi Suzuki

**Affiliations:** ^1^Department of Molecular Therapy, National Institute of Neuroscience, National Center of Neurology and Psychiatry, Tokyo 187-8502, Japan; ^2^Stem Cell Institute, Paul and Sheila Wellstone Muscular Dystrophy Center, Department of Neurology, University of Minnesota Medical School, Minneapolis, MN 55455, USA; ^3^Department of Comparative Biosciences, Stem Cell and Regenerative Medicine Center, University of Wisconsin, Madison, WI 53706, USA

Pluripotent stem cells, which include embryonic stem cells (ESCs) and induced pluripotent stem cells (iPSCs), offer a promising cell source for cell-based therapy to target many degenerative diseases including Duchenne muscular dystrophy (DMD) as well as modeling disease conditions *in vitro*. The proliferation and differentiation properties of ESCs/iPSCs can contribute to preparing a large yield of skeletal muscle stem cells (myogenic progenitors), which is necessary for cell-based therapy. Particularly, iPSC technology allows us to create patient-derived stem cells, which can recapitulate pathophysiological conditions *in vitro*. These *in vitro* disease models are expected to work as a unique platform for drug screening and allow comprehensive studies of disease mechanisms. In addition, patient-derived iPSCs are an especially ideal cell source to obtain an unlimited number of myogenic cells that escape immune rejection after engraftment. In the last decade, a number of culture methods have been published almost annually from different groups for myogenic differentiation from human ESCs/iPSCs. Currently, however, derivation of skeletal muscle stem cells/progenitors with high regenerative potential from human ESCs/iPSCs is still challenging. Further, *in vitro*-generated human ESC/iPSC-derived myofibers are morphologically and functionally less mature than postnatal myofibers.

This special issue was proposed to introduce both comprehensive review articles and an original cutting-edge research article to describe novel insights into skeletal muscle differentiation of ESCs/iPSCs. The topics cover the current status of research progress on ESC/iPSC-based disease modelling and ESC/iPSC-based cell therapy, the technical barriers for the successful induction of skeletal muscle suitable for regenerative medicine and ESC/iPSC-based drug development, and technical tips for *in vitro* disease modeling and ESC/iPSC-based regenerative medicine.

When considering cell-based therapies targeting muscle diseases including DMD, a significant number of myogenic cells with high differentiation quality would be essential. The research article by Y. Ando et al. introduces their current efforts to generate mesenchymal stromal cells for future myoblast therapy from a working cell bank of human ESCs. The authors identified that ESC-derived CD105^+^ cells possess extensive *in vitro* proliferation capability and exhibit efficient myogenic differentiation capacity with genetic stability. Therefore, ESC-derived CD105^+^ cells may be an alternative cell source for myogenic cells in cell-based therapy for patients with genetic muscular disorders.

The review article authored by P. Gee et al. overviews recent advances and applications of the CRISPR/Cas 9 system in the field of stem cell research and how iPSCs and the CRISPR technologies can be applied to gene therapy for DMD. The CRISPR/Cas 9 technology allows us to make precise and targeted editing to the genome of living cells. For DMD, an attractive therapeutic approach is to restore the expression of the dystrophy gene using the CRISPR/Cas 9 system. The authors summarized the current approaches using various CRISPR/Cas 9 strategies to target DMD mutation *in vitro* (patient-derived myoblast and iPSCs) and *in vivo* (DMD mouse model).

The review article by Y. Kodaka et al. summarized the recent protocols of efficient myogenic differentiation using EC/iPSCs. Current approaches of skeletal muscle cell induction of ESCs/iPSCs utilize techniques including overexpression of myogenic transcription factors such as MyoD or Pax3 using small molecules to induce mesodermal cells followed by myogenic progenitor cells. In addition, the authors noticed that epigenetic myogenic memory exists in muscle cell-derived iPSCs, which causes increased myogenic differentiation capacity compared with fibroblast-derived iPSCs. Therefore, muscle cell-derived iPSCs may be utilized for the efficient myogenic differentiation of iPSCs.

Skewed X chromosome inactivation (XCI) is a cause of a severe dystrophic phenotype in female carriers of DMD. Therefore, XCI status in human iPSCs is important for disease modeling of manifesting female DMD carriers. In this special issue, Y. Miyagoe-Suzuki et al. reported frequent reactivation of inactive X chromosomes during iPSC reprogramming. Consequently, many human iPSC clones showed biallelic expression of the androgen receptor (AR) gene and loss of X-inactivation-specific transcript and trimethyl-histone H3 (Lys27) signals on X chromosomes. Particularly, a wild-type dystrophin allele was expressed in multinucleated myotubes differentiated from a manifesting carrier of DMD-iPSCs with a XaXa pattern. Therefore, myotubes differentiated from manifesting female carrier of DMD-iPSCs with two active X chromosomes restored dystrophin expression due to acquirement of nonskewed XCI.

The other review article authored by T. Akiyama et al. presents the roles of epigenetic and transcriptional manipulation for skeletal muscle differentiation from human pluripotent stem cells. Enforced expression of specific myogenic factors such as PAX7 and MYOD1 has been known to promote skeletal muscle differentiation. Current work demonstrates that direct differentiation of human pluripotent stem cells hardly occurs with the ectopic expression of these transcriptional factors due to chromatin features unique to human pluripotent stem cells, which hinder the access of transcription factors to genes involved in muscle differentiation. Recent studies have demonstrated that epigenetic manipulation can enhance myogenic differentiation from human ESCs/iPSCs.

Human iPSCs are a useful tool to investigate the molecular mechanisms of myogenesis in human. K. Higashioka et al. generated MYOGENIN-mutated human iPS cells using CRISPR/Cas9 technology. MYOGENIN is known to function as an essential myogenic transcription factor during the terminal differentiation stage. MYOGENIN gene-knockout mice display deficiency of differentiated skeletal myofibers, while there are residual myofibers in the mutant mice possibly due to functional compensation by MYOD1 and/or MRF4. Interestingly, in the paper, the authors found that human iPSCs can differentiate into skeletal muscle without MYOGENIN activity *in vitro*, indicating similar compensation mechanisms by MYOD1 and/or MRF4 for myogenic differentiation of human iPSCs.

The editors together with the involved authors have discussed the current status of skeletal muscle differentiation using ESCs/iPSCs, the epigenetic alteration occurring during the myogenic reprogramming process, and the related research to expand our knowledge of skeletal muscle differentiation using ESCs/iPSCs. We hope that this special issue can provide valuable information that will further improve protocols for skeletal muscle cell induction from ESCs/iPSCs and that will facilitate the application of ESCs/iPSCs for the future in regenerative medicine of muscle diseases.

*Yuko Miyagoe-Suzuki* *Atsushi Asakura* *Masatoshi Suzuki*

